# The efficacy of therapeutic plasma exchange in COVID-19 patients on endothelial tightness *in vitro* is hindered by platelet activation

**DOI:** 10.3389/fcvm.2023.1094786

**Published:** 2023-05-04

**Authors:** Theo Ebermeyer, Olivier Hequet, Frederic Berard, Amelie Prier, Marie-Ange Eyraud, Charles-Antoine Arthaud, Marco Heestermans, Anne-Claire Duchez, Aurelie Guironnet-Paquet, Philippe Berthelot, Fabrice Cognasse, Hind Hamzeh-Cognasse

**Affiliations:** ^1^ INSERM, U 1059 SAINBIOSE, Université Jean Monnet, Mines Saint-Étienne, F-42023, Saint-Etienne, France; ^2^ Etablissement Français du Sang Auvergne-Rhône-Alpes, Research Department, F-42023, Saint-Etienne, France; ^3^ CIRI-Centre International de Recherche en Infectiologie, Inserm, U1111, CNRS, UMR5308, ENS Lyon, UJM, Université Claude Bernard Lyon 1, Université Jean Monnet de Saint-Etienne, Lyon, France; ^4^ Groupement Hospitalier Sud, Allergy and Clinical Immunology Department, Hospices Civils de Lyon, Lyon, France; ^5^ University Hospital of Saint-Etienne, Infectious Diseases Department, F-42023, Saint-Etienne, France

**Keywords:** COVID-19, therapeutic plasma exchange, endothelium, platelet, inflammation

## Abstract

Coronavirus disease (COVID)-19 is characterised in particular by vascular inflammation with platelet activation and endothelial dysfunction. During the pandemic, therapeutic plasma exchange (TPE) was used to reduce the cytokine storm in the circulation and delay or prevent ICU admissions. This procedure consists in replacing the inflammatory plasma by fresh frozen plasma from healthy donors and is often used to remove pathogenic molecules from plasma (autoantibodies, immune complexes, toxins, etc.). This study uses an *in vitro* model of platelet-endothelial cell interactions to assess changes in these interactions by plasma from COVID-19 patients and to determine the extent to which TPE reduces such changes. We noted that exposure of an endothelial monolayer to plasmas from COVID-19 patients post-TPE induced less endothelial permeability compared to COVID-19 control plasmas. Yet, when endothelial cells were co-cultured with healthy platelets and exposed to the plasma, the beneficial effect of TPE on endothelial permeability was somewhat reduced. This was linked to platelet and endothelial phenotypical activation but not with inflammatory molecule secretion. Our work shows that, in parallel to the beneficial removal of inflammatory factors from the circulation, TPE triggers cellular activation which may partly explain the reduction in efficacy in terms of endothelial dysfunction. These findings provide new insights for improving the efficacy of TPE using supporting treatments targeting platelet activation, for instance.

## Introduction

Severe acute respiratory syndrome coronavirus 2 (SARS-CoV-2) is the infectious agent responsible for Coronavirus Disease 2019 (COVID-19) (1). In some patients, COVID-19 results in acute respiratory distress syndrome and multi-organ failure due in part to the proinflammatory cytokines produced during the immune response, referred as the “cytokine storm”. At cellular level, cytokine-driven leakage of the pulmonary endothelium contributes to COVID-19 pathogeny (2). Indeed, the entry of SARS-CoV-2 within the organism contributes to the inflammatory environment affecting the endothelium and leads to platelet activation and thrombo-inflammation.

Moreover, COVID-19 may also be associated with coagulation disorders including Disseminated Intravascular Coagulation (DIC). In case of COVID-19 related DIC, it is highly associated with mortality and increased levels of pro-thrombotic plasmatic factors including D-dimer levels, and prothrombin time (3–5). Thus, the ISTH interim guidance on recognition and management of coagulopathy in COVID-19 proposed to consider, in case of coagulopathy-related parameters worsening, more “experimental” therapies, including blood product support, in addition to extensive critical care management (6). Yet, it remains unclear whether the dysregulation of thrombotic and coagulation responses stems from SARS-CoV-2 infection *per se* or is related to the “cytokine storm” (7). In patients with severe respiratory failure, immune dysregulation with a macrophage-activation syndrome accompanies this cytokine storm, subsequently followed by immunosuppression. However, the pathophysiolology of COVID-19 is still poorly understood, thus hindering the development of therapeutic strategies and leaving many questions unanswered, particularly on the initiation of inflammation and the subsequent endothelial barrier dysfunction and cardiovascular consequences.

By bridging haemostasis and inflammation, platelets are major players in thrombo-inflammation (8, 9). Platelets are a major source of circulating cytokines, including immunomodulatory molecules involved in cardiovascular diseases, such as soluble CD40-Ligand (sCD40l) (10, 11), which has been proposed to be an early marker of severe acute respiratory syndrome in COVID-19 (12). Moreover, platelets regulate leukocyte and endothelial activities (13, 14), both of which are altered in COVID-19 as reflected, for instance, by increased circulating platelet/leukocyte aggregates (15, 16). It would therefore appear that a COVID-19-related change in platelet-endothelium interactions likely contributes to the severity of COVID-19 (17). Indeed, the hyperactivated state of platelets (18) and endothelial cells (19) during COVID-19 and the related accumulation of inflammatory factors in the circulation amplify pro-inflammatory and pro-thrombotic responses. Thus, a reduction in the circulating levels of inflammatory factors would be an attractive strategy in the early stages of the disease in a bid to prevent complications and alleviate ICU patient management.

Therapeutic Plasma Exchange (TPE) is a procedure that involves replacing patient plasma with fresh frozen plasma from healthy donors obtained through blood donation or by plasmapheresis. This treatment is already used for patients with an harmful accumulation of blood molecules such as IgMs in Waldenström's macroglobulinemia or autoantibodies in ANCA-associated vasculitis (20). Successive sessions of TPE remove these molecules in order to improve the patient's condition. This treatment is considered effective, well tolerated and safe. The main advantage of this approach is that it triggers a significant, albeit transient, decrease in thrombo-inflammatory blood molecules without inducing immunosuppression. If implemented in the early stages, this strategy could improve thrombo-inflammation in patients with severe COVID-19 by replacing inflammatory molecules and accumulated activated haemostatic factors with healthy plasma components, thereby delaying or preventing ICU admission (21). The ongoing Therapeutic Plasma Exchange to Alleviate the Hyperinflammatory Condition During Severe Covid-19 Infections (CovidEP) trial (ClinicalTrials Identifier: NCT04751643) addresses this issue and assesses the requirement for intubation and invasive ventilation in patients undergoing 3 TPE sessions in 3 consecutive days compared to patients receiving standard treatments including assisted ventilation, oxygen supplementation, non-invasive ventilation, vasopressive support and corticosteroids, etc. In parallel to this clinical study, the results of which are not available yet, it has been proposed to evaluate *in vitro* the inflammatory potential of the plasma of patients undergoing TPE vs. those receiving standard therapy. Plasma samples from COVID-19 patients, prior to either treatment, alter endothelial tightness and barrier function. TPE had a beneficial impact on the endothelium, which was slightly reduced in the presence of platelets, whose activation led to that of endothelial cells. Our encouraging results suggest that TPE may contribute to an improvement in COVID-19-related complications, including endothelial dysfunction.

## Method

### Patient samples and ethical considerations

In accordance with the French Public Health Code (Article L. 1223-3) and the procedures of the French Blood Bank Ethics Board (Etablissement Français du Sang—EFS), volunteer blood donors signed the donation form indicating that they did not object to the use of their donation for non-therapeutic purposes. Thus, in accordance with the Declaration of Helsinki, informed and written consent has been obtained from all the healthy donors who participated in this study for blood sampling for scientific purposes.

Plasma samples from hospitalised COVID-19 adults were obtained during the CovidEP clinical trial (NCT04751643) upon information and with the written consent of the patient or, if this was not possible, from a trusted individual. COVID-19 patients were randomized in two groups, a control group (*n* = 10) receiving standard treatments (assisted ventilation, oxygen supplementation, non-invasive ventilation, invasive ventilation, antibiotic, vasopressive support or corticosteroids) and a TPE group (*n* = 10) receiving standard treatments and, 24 h after inclusion, three consecutive TPE sessions, i.e., one per day for 3 consecutive days. TPE was performed using a centrifugation technique (Optia TerumoBCT, Lakewood, CO, USA). This procedure consisted of an exchange of 1.2 plasma volumes. The plasma removed was replaced by fresh-frozen plasma (collected before the COVID-19 pandemic). In all sessions dual vascular access and acid citrate dextrose-A (ACD-A) were used. Inlet Blood was mixed with ACD-A in ratio of 17:1. Continuous infusion of calcium and magnesium on outlet blood was performed to avoid ACD-A toxicity. Plasma sampling was performed on patients in both groups on inclusion day (Day 0, D0) and therefore before TPE for patients in this group and 4 days after inclusion (D4), i.e., 24 h after the 3rd TPE session for this group of patients. Plasma was prepared according to guidelines from the French Study Group on Haemostasis and Thrombosis. Briefly, citrated blood samples were centrifuged for 10 min at 2,000–2,500 g. Plasma samples were stored at −80°C until required for subsequent analysis. Patient characteristics and comorbidities are listed in Supplementary Table S1. The COVID-19 TPE patients and COVID-19 controls groups were matched by gender and, where possible, age, with the limitation of the number of samples available for the study.

### Chemicals and reagents

Dulbecco's Modified Eagle's Medium—High-Glucose (DMEM-HG), MEM Non-essential Amino Acid Solution, Antibiotic Antimycotic Solution, Dulbecco's Phosphate Buffered Saline (PBS), Apyrase, Prostaglandin I2 sodium salt (PGI2), Trypsin-EDTA and Fluorescein Isothiocyanate (FITC)-Dextran 4 kDa were purchased from Sigma-Aldrich (Saint-Quentin-Fallavier, France). Hank's Balanced Salt Solution (HBSS), HEPES Buffer Solution and cell culture inserts for 24-well plates [high-density pore, 3 µm pore diameter, translucent poly terephthalate ethylene (PET) membrane] were purchased from Thermo Fischer Scientific/Fisher Scientific S.A.S. (Illkirch, France). Foetal calf serum was purchased from Eurobio Scientific (Les Ullis, France). ThromboFix Platelet Stabiliser was purchased from Beckman Coulter (Villepinte, France). Allophycocyanin (APC)-conjugated mouse anti-human CD41a (clone HIP-8), Phycoerythrin (PE)-conjugated mouse anti-human CD54 (clone HA58), PE-CD62P (clone AK4), FITC-CD63 (clone H5C6) and fluorochrome-conjugated mouse IgG isotypic controls were purchased from BD Biosciences (Le Pont de Claix, France).

### Cell culture and creation of an *in vitro* endothelial cell barrier model

Human endothelial cell line EA.hy926 was purchased from the American Type Culture Collection/ LGC Standards SARL (Molsheim, France). EA.hy926 endothelial cells (ECs) were cultured in DMEM-HG supplemented with heat-inactivated foetal calf serum (10% v/v), MEM (1% v/v) and antibiotic-antimycotic solution (1% v/v) and maintained at 37°C in a 5% carbon dioxide atmosphere. On reaching 80% confluence, ECs were subcultured using 0.5% trypsin-EDTA solution.

A tight monolayer was obtained on translucent PET using 3 µm pore-sized culture inserts with a 0.33 cm^2^ growth area. Briefly, ECs were seeded at a density of 10^5^ cells/wells and grown for 18 days in order to reach optimal barrier properties, reflected by maximal Trans-Endothelial Electrical Resistance (TEER).

### Platelet preparation

Peripheral blood was collected from healthy donors in endotoxin-free 3.8% sodium citrate tubes (Vacutainer®, Becton-Dickinson) and whole blood tubes were stored at 37°C before use and within two hours of collection. Platelet-rich plasma (PRP) was prepared by centrifuging blood at 188 ×  g for 10 min at room temperature. Platelet counts were carried out in freshly prepared PRP using an MS4s Haematology analyser (Melet Schloesing, Osny, France) and a sufficient volume of PRP to obtain 2 × 10^7^ platelets for each co-culture was centrifuged at 423 × g for 10 min at RT. The cell pellet was then resuspended in 500 µl of D0 or D4 plasma from COVID-19 patients in either the control or TPE group and used immediately.

### Co-culture of platelets and endothelial cells

EC monolayers on Transwell inserts were either incubated with COVID-19 D0 or D4 plasma alone, or co-cultured with healthy platelets as described previously (22) from PRP prepared according to the protocol described in the paragraph above, centrifugated and resuspended in COVID-19 D0 or D4 plasma for 24 h at 37°C and 5% CO2.

Each patient plasma, when combined with healthy donor platelets, was evaluated in the presence of platelets from two independent donors. Each data point corresponds to an individual data point. Endothelial barrier integrity was then assessed and cell supernatants were stored at −80°C until required for the inflammatory cytokine assessment. Platelets and ECs were analysed by flow cytometry to assess their activation status.

### Expression of platelet and endothelial activation markers

Platelet membrane expression of *P*-selectin (CD62P) and CD63 activation markers following the co-culture of platelet and endothelial cells was assessed after immunostaining of platelets with APC-CD41a (for gating strategy), PE-CD62P and FITC-CD63 or corresponding isotypic controls. Platelets were fixed with ThromboFix prior to flow cytometry analysis. ICAM-1/CD54 membrane expression was assessed on ECs by immunostaining after trypsinization and cell washing. ECs were fixed in 2% paraformaldehyde which was replaced with PBS before the flow cytometry analyses. The analyses were performed either on a BD CANTO II flow cytometer and analysed with FlowJo V10.8 (Ashland, USA) software or on a BD Guava® easyCyte™ 8HT device and analysed with BD FACS Diva™ software (BD Biosciences). Expression of activation markers by EC exposed to COVID-19 plasmas were reported to those assessed on EC cultured with medium, as control. Activation marker levels of platelets cocultured with EC and exposed to COVID-19 plasmas were reported to those assessed on platelets before co-culture. The expression levels of activation markers after exposure to COVID-19 plasmas were expressed as fold increase.

### Evaluation of endothelial barrier properties

Endothelial permeability was assessed using FITC-Dextran. HBSS transporter buffer supplemented with 10 mM HEPES containing 10 µg/ml FITC-Dextran was loaded on the apical side of the insert and incubated at 37°C for 1 h. Fluorescence was measured in the basolateral compartment using a Tecan Infinite® 200Pro fluorescence spectrophotometer (Männedorf, Switzerland) with 485 nm excitation and 530 nm emission wavelengths. Dextran passage to the basolateral compartment is expressed as a percentage of the total fluorescence deposited in the apical compartment, measured at the beginning of the incubation.

TEER was recorded using an EVOM®resistance meter with STX-2 electrode (Sarasota, USA) to characterize the formation of a tight endothelial cell monolayer on Transwells. The measurement of a blank insert, i.e., without cells, was performed and the signal was subtracted from that recorded for the insert with cells. The resulting value was then reported at the membrane area (0.3 cm^2^) to obtain the TEER measurement in Ω.cm².

### Inflammatory factor quantification

The amount of inflammatory factors in culture supernatants was measured using Luminex technology or specific ELISA. ELISA was used to quantify RANTES. Luminex technology was used to measure sCD40l, sCD62P and interleukin (IL)-6. Luminex and ELISA data were expressed in pg/ml. In order to assess molecule production during ECs and platelet co-culture on COVID-19 plasma stimulation, the quantity of each factor was assessed in COVID-19 D0 and D4 plasma and then subtracted from the amount assessed in culture supernatants.

### Statistical analysis

Statistical analysis was performed using GraphPad Prism 8 software (San Diego, USA). Statistical significance was defined as 2-sided *P* value of less than.05 and unadjusted for multiple comparisons. Data were expressed as the point estimate of mean ± the margin of error around the mean defining the 95% confidence interval. The non-parametric Wilcoxon matched-pairs signed rank test was used to assess the significance of parameter changes over time. *p* < 0.05 was considered significant.

## Results

### TPE reduces endothelial leakage caused by COVID-19 plasma

**Figure 1:**
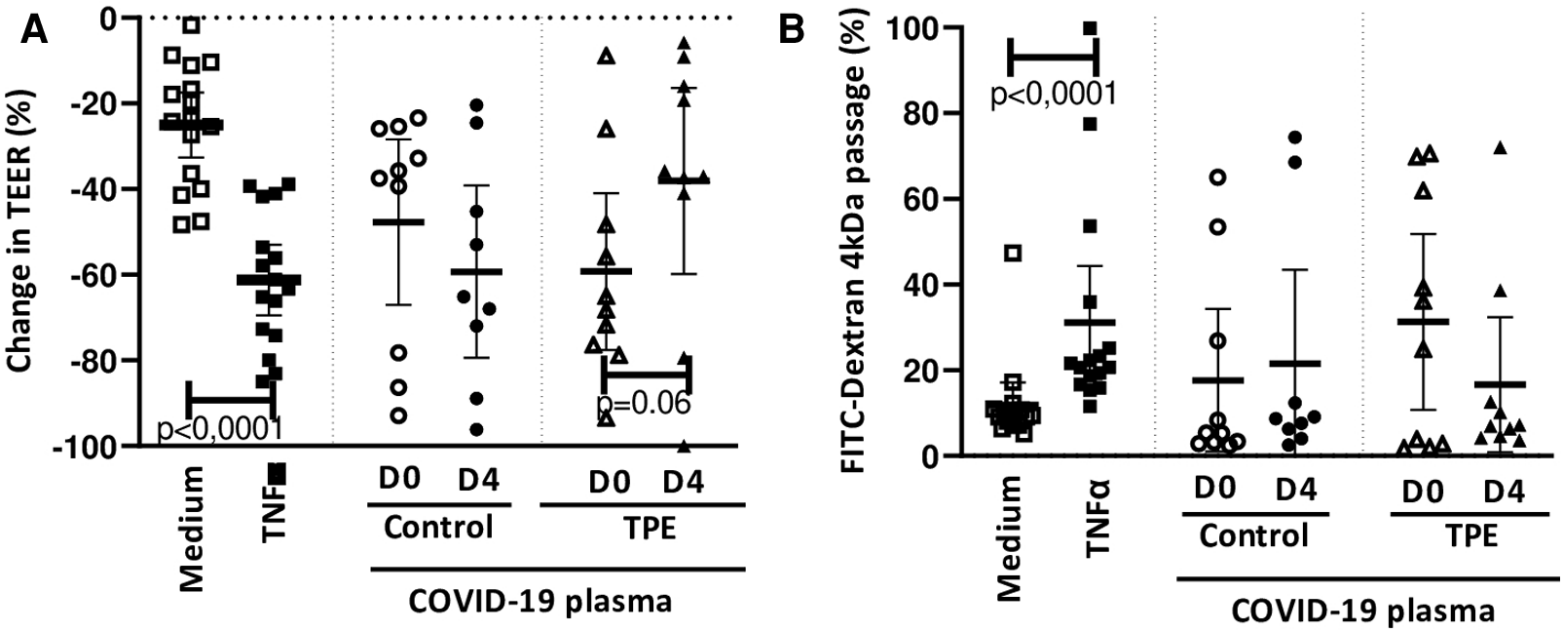
Effect of COVID-19 control and TPE plasma on endothelial monolayer tightness. TEER (**A**) and FITC-Dextran 4 kDa passage (**B**) were assessed on an endothelial cell monolayer exposed for 24 h to culture medium as a negative control (*n* = 16), 100 ng/ml TNF*α* as a positive control (*n *= 16), COVID-19 D0 or D4 control or TPE plasma (*n* = 9–10). (**A**) Relative TEER was expressed as a percentage change in relation to TEER assessed prior to stimulation. (**B**) FITC-Dextran 4 kDa passage through the endothelial monolayer was expressed as a percentage of the total fluorescence deposited in the apical compartment. Data are presented as individual values with the point estimate of mean ± the margin of error around the mean defining the 95% confidence interval. The non-parametric Wilcoxon matched-pairs signed rank test was used to assess the significance of changes in parameters over time. *p* < 0.05 was considered significant.

We observed that 24 h exposure of the endothelial monolayer to COVID-19 D0 plasma from either the control or the TPE group triggered an important reduction in TEER, amounting to 47.7 ± 19% and 59.2 ± 18%, respectively (Figure 1A). This TEER reduction was higher than the natural TEER decrease occurring on culturing with the medium for an additional 24 h (25.0 ± 8%, Figure 1A) and almost reached that observed on endothelial stimulation with 100 ng/mL TNF*α* (61.1 ± 10%, Figure 1A). Moreover, upon exposure of the endothelium to COVID-19 D4 control plasmas, the decrease in TEER value was even more dramatic (59.2 ± 20%), whereas COVID-19 D4 TPE plasma had obviously a much lesser impact on TEER (38.0 ± 22%, *p* = 0.06, Figure 1A). In order to address whether this drop in TEER values led to increased endothelial permeability, we then assessed the passage of FITC-Dextran 4 kDa molecules though the monolayer exposed to COVID-19 plasma. We were able to show that COVID-19 control plasma mediated Dextran passage through the endothelium increased from D0 to D4, with values ranging from 17.6 ± 17% to 21.5 ± 24% for COVID-19 control D0 and D4 plasma, respectively (Figure 1B). This was higher than the value observed with the culture medium (11.8 ± 6%, Figure 1B). Conversely, exposure of the endothelium to COVID-19 TPE plasma resulted in a decrease, though not statistically significant (*p* = 0,23), in endothelial leakage from 31.3 ± 16% observed for D0 TPE plasma to16.6 ± 22% for D4 TPE plasma, which may suggest that D4 TPE plasma does not alter endothelial permeability more than COVID-19 D4 control plasma.

### Platelets co-cultured with endothelium alter endothelial permeability in the presence of COVID-19 plasma

**Figure 2:**
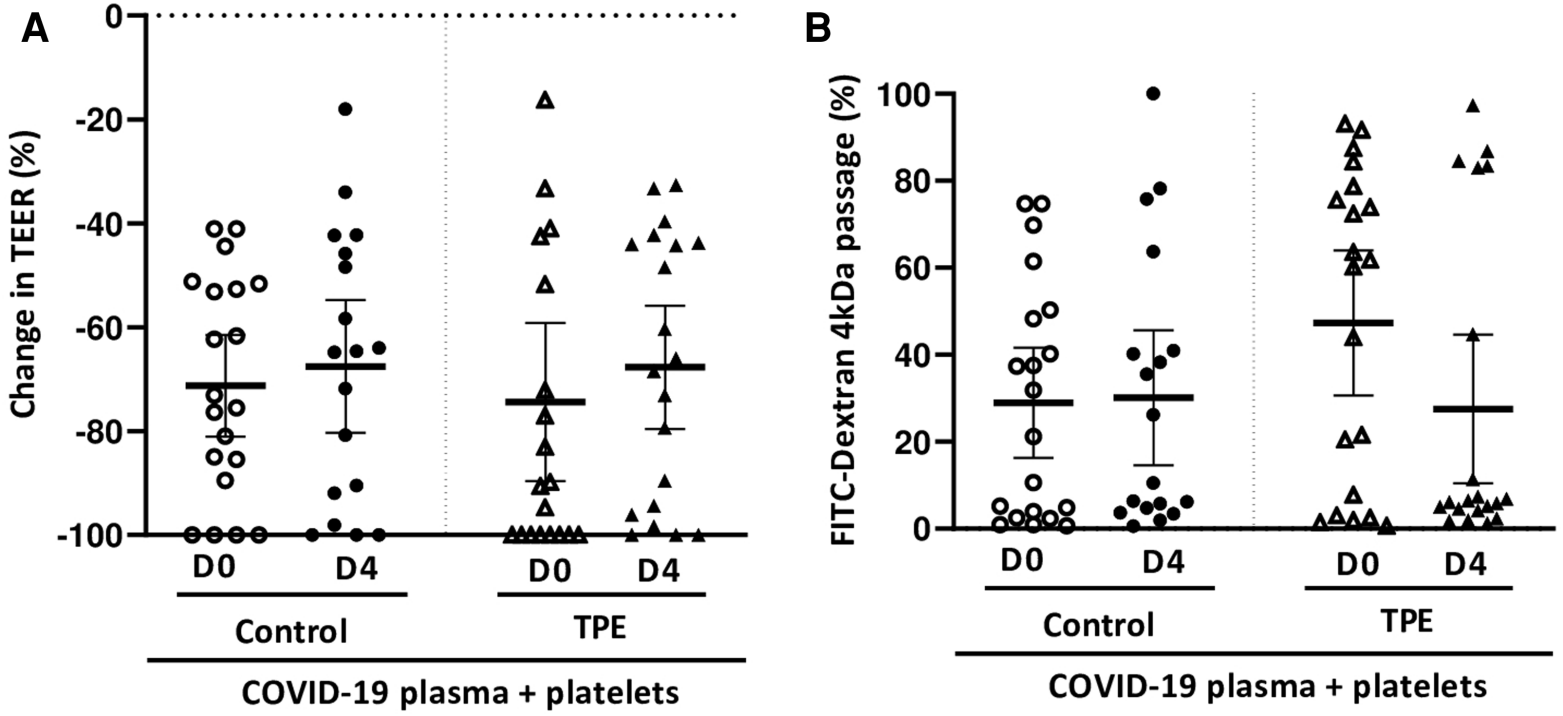
Effect of COVID-19 control and TPE plasma on endothelial tightness in a platelet/endothelial co-culture. TEER (**A**) and FITC-Dextran 4 kDa passage (**B**) were assessed on an endothelial cell monolayer co-cultured with healthy platelets and exposed for 24 h to COVID-19 D0 or D4 control or TPE plasma (*n* = 9−10, each plasma tested with platelets from two independent donors). (**A**) Relative TEER was expressed as a percentage change in relation to TEER assessed prior to incubation. (**B**) FITC-Dextran 4 kDa passage through the endothelial monolayer was expressed as a percentage of the total fluorescence deposited in the apical compartment. Data are presented as individual values with the point estimate of mean ± the margin of error around the mean defining the 95% confidence interval. The non-parametric Wilcoxon matched-pairs signed rank test was used to assess the significance of changes in parameters over time. *p* < 0.05 was considered significant.

In order to resemble physiological conditions more closely, healthy platelets were added to the endothelial monolayer and the whole was co-cultured for 24 h in the presence of COVID-19 plasma either from the control or the TPE group. We found that platelet addition worsened the TEER decrease induced by COVID-19 plasma to a similar extent for control and TPE plasma. Indeed, in the presence of platelets, COVID-19 D0 plasma triggered TEER decreases of 71.2 ± 11% and 74.3 ± 15% for the control and TPE plasma, respectively (Figure 2A). Similarly, COVID-19 D4 control and TPE plasma induced comparable TEER decreases of 67.5 ± 14% and 67.6 ± 12%, respectively, in platelet-endothelium co-culture. This reduction in TEER values observed in platelet-endothelium co-culture on COVID-19 plasma stimulation was also accompanied by an increase in endothelial permeability in the presence of either the control or TPE plasma (Figure 2B). Although Dextran passage reached high and stable levels in the presence of platelets on D0 (29.0 ± 12%) and D4 (30.1 ± 16%) control plasma stimulation, a different pattern was displayed for TPE plasmas (Figure 2B). In fact, endothelial permeability was greatly enhanced by COVID-19 D0 TPE plasma (47.3 ± 15%), but stimulation of platelet-endothelium co-culture with D4 TPE plasma resulted in a 27.5 ± 18% decrease in Dextran passage (Figure 2B).

### Platelet activation in the presence of TPE plasma

**Figure 3:**
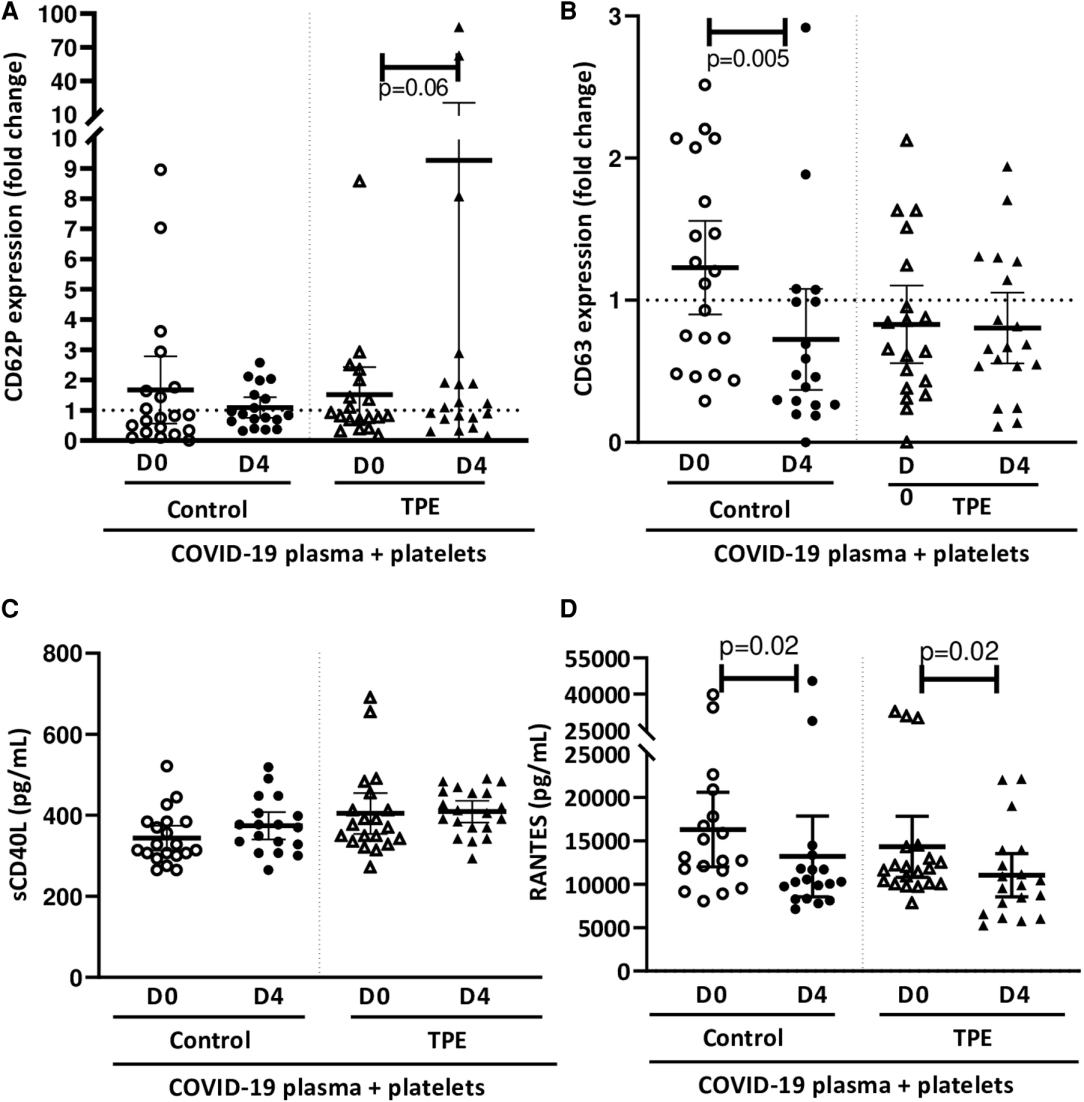
Effect of COVID-19 control and TPE plasma on platelet activation in a platelet/endothelial co-culture. Membrane expression of CD62P (**A**) and CD63 (**B**) and release of sCD40l (**C**) and RANTES (**D**) by platelets were assessed following 24 h-exposure of the endothelial cell monolayer co-cultured with healthy platelets to COVID-19 D0 or D4 control or TPE plasma for 24 h (*n* = 9−10, each plasma tested with platelets from two independent donors, each data point corresponds to an individual data point). Relative CD62P and CD63 platelet expression was expressed as a ratio (fold change) in relation to the co-culture exposed to the culture medium. The release of sCD40l and RANTES by platelets following exposure to COVID-19 plasma was expressed in pg/mL and calculated by subtracting the amount of these factors present in plasma from the total amount assessed in the co-culture supernatant after stimulation. Data are presented as individual values with the point estimate of mean ± the margin of error around the mean defining the 95% confidence interval. The non-parametric Wilcoxon matched-pairs signed rank test was used to assess the significance of changes in parameters over time. *p* < 0.05 was considered significant.

Observations on how platelets altered the endothelial barrier in the presence of COVID-19 prompted us to investigate platelet and endothelial activation. The increase in CD62P (Figure 3A) and CD63 (Figure 3B) membrane expression on platelets co-cultured with the endothelium exposed to COVID-19 plasma appeared to be quite moderate. Indeed, CD62P expression by platelets on exposure to D0 and D4 control plasma resulted in 1.7 ± 1.2 and 1.1 ± 0.4-fold increases, respectively. However, platelets co-cultured with endothelial cells in the presence of D4 TPE plasma were strongly activated, as reflected by the 9.3 ± 11.6-fold increase in CD62P expression (Figure 3A). However, CD63 expression by platelets co-cultured on the endothelial monolayer did not appear to be affected by COVID-19 plasma, except for D4 control plasma, which triggered a 0.7 ± 0.4-fold decrease in this marker (*p* = 0.005).

Interestingly, platelets did not release significant amount of sCD40l in co-culture with the endothelium further to any COVID-19 plasma stimulation, even for that triggering significant CD62P expression (Figure 3C). In contrast, RANTES production by platelets in co-culture seemed to be affected by COVID-19 plasma. Indeed, a significantly lower quantity of RANTES was released by platelets on exposure to D4 plasma (13,213 ± 5,600 pg/ml for control plasma and 11,046 ± 3,200 pg/ml for TPE plasma), compared to D0 plasma (16,291 ± 4,800 pg/ml for control plasma and 14,304 ± 4,000 pg/ml for TPE plasma, D0 vs. D4 *p* = 0.02), irrespective of the patient group (Figure 3D).

### Moderate endothelial activation is related to platelet activation

**Figure 4:**
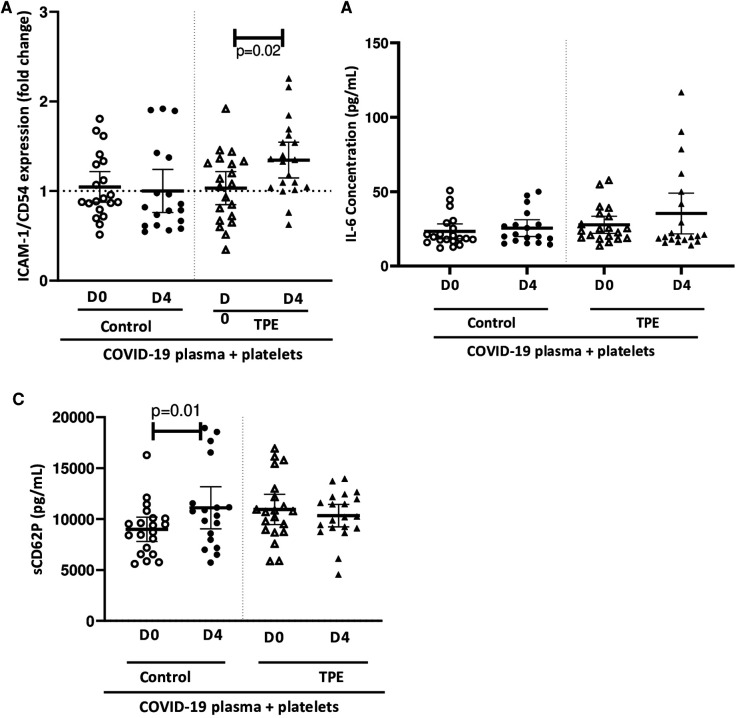
Effect of COVID-19 control and TPE plasma on endothelial activation in a platelet/endothelial co-culture. Membrane expression of ICAM-1/CD54 (**A**) and release of IL-6 (**B**) and sCD62P (**C**) by endothelial cells were assessed following 24 h-exposure of the endothelial cell monolayer co-cultured with healthy platelets to COVID-19 D0 or D4 control or TPE plasma for 24 h (*n* = 9−10, each plasma tested with platelets from two independent donors, each data point corresponds to an individual data point). Relative ICAM-1/CD54 expression by endothelial cells was expressed as a ratio (fold change) in relation to the co-culture exposed to the culture medium. The release of IL-6 and sCD40l by endothelial cells following exposure to COVID-19 plasma was expressed in pg/ml and calculated by subtracting the amount of these factors present in plasma from the total amount assessed in the co-culture supernatant after stimulation. Data are presented as individual values with the point estimate of mean ± the margin of error around the mean defining the 95% confidence interval. The non-parametric Wilcoxon matched-pairs signed rank test was used to assess the significance of changes in parameters over time. *p* < 0.05 was considered significant.

In parallel to platelet activation, we evaluated endothelial cell activation and assessed the expression of ICAM-1/CD54 on co-culture with platelets and exposure to COVID-19 plasma. None of the COVID-19 control plasma induced ICAM-1 expression by endothelial cells co-cultured with platelets, whereas only the D4 TPE plasma induced a significant overexpression of this marker (1.3 ± 0.2-fold increase, *p* = 0.02,

Figure 4A). Since TPE plasmas used alone failed to induce ICAM-1 overexpression (Supplementary Figure S1), the ICAM-1 overexpression by endothelial cells on exposure to D4 TPE plasma that was observed on Figure 4A could be related to the presence of platelets.

Similarly, no COVID-19 plasma exposure triggered significant endothelial IL-6 production, which ranged from 23.3 ± 8.3 pg/ml to 25.6 ± 6.3 pg/ml for control plasma and 27.7 ± 10.4 pg/ml to 35.4 ± 20.8 pg/ml for TPE plasma (Figure 4B). The same observation was made for sCD62P except on stimulation of the co-culture with D4 control plasma, which induced a slightly higher sCD62P production compared to stimulation with D0 control plasma, due either to endothelial cells or platelets since both cell types release this molecule (8,895 ± 1,154 pg/ml for D0 control plasma and 11,103 ± 1,923 pg/ml for D4 control plasma, *p* = 0.01, Figure 4C).

## Discussion

In using an *in vitro* culture model comprising a tight endothelial monolayer, this study shows that TPE improves the inflammatory component of patient plasma compared to that of patients receiving conventional treatments. First and foremost, endothelial barrier properties reflected by TEER and Dextran passage were altered on exposure to COVID-19 D0 plasma from both groups, i.e., plasma collected at baseline, prior to treatment. While endothelial permeability increased in the presence of control group D4 plasma, it improved substantially in the presence of plasma collected from patients post-TPE. Indeed, TPE has been shown to be particularly interesting in sepsis, which triggers a huge cytokine storm in the same way as COVID-19. Knaupp et al. have elegantly demonstrated that early TPE reduced both the medication requirement and circulating levels of pro-inflammatory cytokines (23). They demonstrated *ex vivo* with cultured endothelial cells that patient plasma triggered less endothelial permeability after TPE than before. Their observations are entirely in line with ours. However, they evidenced some variability between plasma samples with a proportion of plasma triggering a comparable increase in endothelial permeability both before and after TPE. This variation in results was also apparent in our study particularly when our system became more complex following the addition of healthy platelets to the endothelial culture.

Thus, the exposure of platelets co-cultured with endothelial cells to COVID-19 D4 TPE plasma induced membrane overexpression of platelet and endothelial activation markers, namely CD62P and ICAM-1, respectively. This was not apparent with COVID-19 control plasma—hence, although the TPE procedure removes inflammatory factors from the patient's circulation, three successive TPE-related apheresis procedures may trigger, to some extent, a cellular activation resulting, four days later, in the accumulation of substances inducing endothelial and platelet activation. This hypothesis is supported by observations showing that TPE affects immune cells, particularly T cells, with a shift in the Th1/2 response towards Th2 (24, 25), potentially limiting the beneficial effect of TPE.

Nevertheless, the phenotypical activation of platelets and endothelial cells that we observed following exposure to COVID-19 TPE plasma did not lead to the production of platelet or endothelial inflammatory factors such as sCD40l or IL-6, nor to any increase in endothelial permeability, which was comparable to that observed on exposure of the platelet-endothelial co-culture to COVID-19 control plasma. However, given the small number of patients in our study, these observations should be confirmed in larger cohorts.

Our results illustrate the complex interplay between platelets and the endothelium in the inflammatory context related to COVID-19. Some studies have showed that, during COVID-19, hyper-activated and hyper-responsive platelets induce endothelial activation, triggering genes involved in coagulation and inflammation at both transcriptomic and proteomic levels (18, 26, 27). In turn, the activated endothelium fuels reciprocal platelet activation and creates a vicious circle sustaining thrombo-inflammation. TPE aims to break this circle by removing the patient plasma containing all of the inflammatory factors and replacing it with fresh frozen plasma. This procedure has already been used during the COVID-19 pandemic with inconsistent results. Indeed, some studies highlighted a beneficial outcome on mortality (28) and others an improvement in terms of clinical features (29, 30) without any obvious impact on mortality (31, 32). However, very few studies investigated cellular responses to TPE and were limited to sepsis (23). Thus, further controlled, prospective studies are required to assess the advantage of using TPE to treat COVID-19 patients. These studies should be conducted alongside analyses of the effects of plasma on cellular responses in a bid to identify the limitations of this procedure. Our results suggest that TPE may be associated with platelet and endothelial activation, which could affect endothelial integrity. An interesting prospect would be to evaluate the use of drugs targeting platelet activation as an adjunct to TPE or to use convalescent COVID-19 plasma instead of fresh frozen plasma from healthy donors.

## Data Availability

The raw data supporting the conclusions of this article will be made available by the authors, without undue reservation.

## References

[1] Zhou P Yang XL Wang XG Hu B Zhang L Zhang W A pneumonia outbreak associated with a new coronavirus of probable bat origin. Nature. (2020) 579:270–3. 10.1038/s41586-020-2012-7 32015507 PMC7095418

[2] Gustafson D Raju S Wu R Ching C Veitch S Rathnakumar K Overcoming barriers: the endothelium as a linchpin of coronavirus disease 2019 pathogenesis? Arterioscler Thromb Vasc Biol. (2020) 40:1818–29. 10.1161/ATVBAHA.120.314558 32510978 PMC7370857

[3] Tang N Li D Wang X Sun Z. Abnormal coagulation parameters are associated with poor prognosis in patients with novel coronavirus pneumonia. J Thromb Haemost. (2020) 18:844–7. 10.1111/jth.14768 32073213 PMC7166509

[4] Arachchillage DR Laffan M. Abnormal coagulation parameters are associated with poor prognosis in patients with novel coronavirus pneumonia. J Thromb Haemost. (2020) 18:1233–4. 10.1111/jth.14820 32291954 PMC7262191

[5] Zhou F Yu T Du R Fan G Liu Y Liu Z Clinical course and risk factors for mortality of adult inpatients with COVID-19 in Wuhan, China: a retrospective cohort study. Lancet. (2020) 395:1054–62. 10.1016/S0140-6736(20)30566-3 32171076 PMC7270627

[6] Thachil J Tang N Gando S Falanga A Cattaneo M Levi M ISTH Interim guidance on recognition and management of coagulopathy in COVID-19. J Thromb Haemost. (2020) 18:1023–6. 10.1111/jth.14810 32338827 PMC9906133

[7] Huang C Wang Y Li X Ren L Zhao J Hu Y Clinical features of patients infected with 2019 novel coronavirus in Wuhan, China. Lancet. (2020) 395:497–506. 10.1016/S0140-6736(20)30183-5 31986264 PMC7159299

[8] D’ Atri L Schattner M. Platelet toll-like receptors in thromboinflammation. Front Biosci (Landmark Ed). (2017) 22:1867–83. 10.2741/4576 28410150

[9] Guo L Rondina MT. The era of thromboinflammation: platelets are dynamic sensors and effector cells during infectious diseases. Front Immunol. (2019) 10:2204. 10.3389/fimmu.2019.02204 31572400 PMC6753373

[10] Morrell CN Aggrey AA Chapman LM Modjeski KL. Emerging roles for platelets as immune and inflammatory cells. Blood. (2014) 123:2759–67. 10.1182/blood-2013-11-462432 24585776 PMC4007605

[11] Cognasse F Laradi S Berthelot P Bourlet T Marotte H Mismetti P Platelet inflammatory response to stress. Front Immunol. (2019) 10:1478. 10.3389/fimmu.2019.01478 31316518 PMC6611140

[12] Hamzeh-Cognasse H Mansour A Reizine F Mismetti P Gouin-Thibault I Cognasse F. Platelet-derived sCD40l: specific inflammatory marker for early-stage severe acute respiratory syndrome coronavirus 2 infection. Virol J. (2021) 18:211. 10.1186/s12985-021-01680-3 34715884 PMC8554745

[13] Hamzeh-Cognasse H Cognasse F Palle S Chavarin P Olivier T Delezay O Direct contact of platelets and their released products exert different effects on human dendritic cell maturation. BMC Immunol. (2008) 9:54. 10.1186/1471-2172-9-54 18817542 PMC2564901

[14] Ho-Tin-Noe B Demers M Wagner DD. How platelets safeguard vascular integrity. J Thromb Haemost. (2011) 9(Suppl 1):56–65. 10.1111/j.1538-7836.2011.04317.x 21781242 PMC3229170

[15] Hottz ED Azevedo-Quintanilha IG Palhinha L Teixeira L Barreto EA Pão CRR Platelet activation and platelet-monocyte aggregate formation trigger tissue factor expression in patients with severe COVID-19. Blood. (2020) 136:1330–41. 10.1182/blood.2020007252 32678428 PMC7483437

[16] Manne BK Denorme F Middleton EA Portier I Rowley JW Stubben C Platelet gene expression and function in patients with COVID-19. Blood. (2020) 136:1317–29. 10.1182/blood.2020007214 32573711 PMC7483430

[17] Jose RJ Manuel A. COVID-19 cytokine storm: the interplay between inflammation and coagulation. Lancet Resp Med. (2020) 8:e46–7. 10.1016/S2213-2600(20)30216-2 PMC7185942 32353251

[18] Zaid Y Puhm F Allaeys I Naya A Oudghiri M Khalki L Platelets can associate with SARS-cov-2 RNA and are hyperactivated in COVID-19. Circ Res. (2020) 127:1404–18. 10.1161/CIRCRESAHA.120.317703 32938299 PMC7641188

[19] Bonaventura A Vecchié A Dagna L Martinod K Dixon DL Van Tassell BW Endothelial dysfunction and immunothrombosis as key pathogenic mechanisms in COVID-19. Nat Rev Immunol. (2021) 21:319–29. 10.1038/s41577-021-00536-9 33824483 PMC8023349

[20] Padmanabhan A Connelly-Smith L Aqui N Balogun RA Klingel R Meyer E Guidelines on the use of therapeutic apheresis in clinical practice—evidence-based approach from the writing committee of the American society for apheresis: the eighth special issue. J Clin Apher. (2019) 34:171–354. 10.1002/jca.21705 31180581

[21] Prakash S Sahu A Routray SS Maiti R Mitra JK Mukherjee S. Efficacy of therapeutic plasma exchange in severe COVID-19 disease: a meta-analysis. Vox Sang. (2022) 118:49–58. 10.1111/vox.13367 PMC9874931 36254849

[22] Proença-Ferreira R Brugnerotto AF Garrido VT Dominical VM Vital DM Ribeiro Mde F Endothelial activation by platelets from sickle cell anemia patients. PLoS One. (2014) 9:e89012. 10.1371/journal.pone.0089012 24551209 PMC3923877

[23] Knaup H Stahl K Schmidt BMW Idowu TO Busch M Wiesner O Early therapeutic plasma exchange in septic shock: a prospective open-label nonrandomized pilot study focusing on safety, hemodynamics, vascular barrier function, and biologic markers. Crit Care. (2018) 22:285. 10.1186/s13054-018-2220-9 30373638 PMC6206942

[24] Goto H Matsuo H Nakane S Izumoto H Fukudome T Kambara C Plasmapheresis affects T helper type-1/T helper type-2 balance of circulating peripheral lymphocytes. Ther Apher. (2001) 5:494–6. 10.1046/j.1526-0968.2001.00386.x 11800088

[25] Kambara C Matsuo H Fukudome T Goto H Shibuya N. Miller fisher syndrome and plasmapheresis. Ther Apher. (2002) 6:450–3. 10.1046/j.1526-0968.2002.00466.x 12460409

[26] Barrett TJ Cornwell M Myndzar K Rolling CC Xia Y Drenkova K Platelets amplify endotheliopathy in COVID-19. Sci Adv. (2021) 7:eabh2434. 10.1126/sciadv.abh2434 34516880 PMC8442885

[27] Dechamps M De Poortere J Martin M Gatto L Daumerie A Bouzin C Inflammation-Induced coagulopathy substantially differs between COVID-19 and septic shock: a prospective observational study. Front Med (Lausanne). (2021) 8:780750. 10.3389/fmed.2021.780750 35111777 PMC8801505

[28] Qin J Wang G Han D. Benefits of plasma exchange on mortality in patients with COVID-19: a systematic review and meta-analysis. Int J Infect Dis. (2022) 122:332–6. 10.1016/j.ijid.2022.06.014 35709964 PMC9192121

[29] Al-Hashami S Khamis F Al-Yahyay M Al-Dowaiki S Al-Mashaykhi L Al-Khalili H Therapeutic plasma exchange: a potential therapeutic modality for critically ill adults with severe acute respiratory syndrome coronavirus 2 infection. J Clin Apher. (2022) 37:563–72. 10.1002/jca.22011 36102158 PMC9538054

[30] Jamil Z Khan AA Yousuf H Khalid K Abbasi SM Waheed Y. Role of therapeutic plasmapheresis in SARS-CoV-2 induced cytokine release syndrome: a retrospective cohort study on COVID-19 patients. Int J Gen Med. (2022) 15:4907–16. 10.2147/IJGM.S362151 35585996 PMC9109892

[31] Cegolon L Einollahi B Panahi Y Imanizadeh S Rezapour M Javanbakht M On therapeutic plasma exchange against severe COVID-19-associated pneumonia: an observational clinical study. Front Nutr. (2022) 9:809823. 10.3389/fnut.2022.809823 35308291 PMC8926159

[32] Diskin CJ Maldonado R Leon J Dansby LM Carter TB Radcliff L How effective is rescue therapeutic plasma exchange in treatment of SARS-coronavirus-2? Ther Apher Dial. (2022) 27:170–6. 10.1111/1744-9987.13862 35490343 PMC9348252

