# Supplementary figures and images for: The efficacy of therapeutic plasma exchange in COVID-19 patients on endothelial tightness in vitro is hindered by platelet activation
Source: Front Cardiovasc Med. 2023 May 4;10:1094786. doi: 10.3389/fcvm.2023.1094786 (PMC10192624; doi:10.3389/fcvm.2023.1094786)

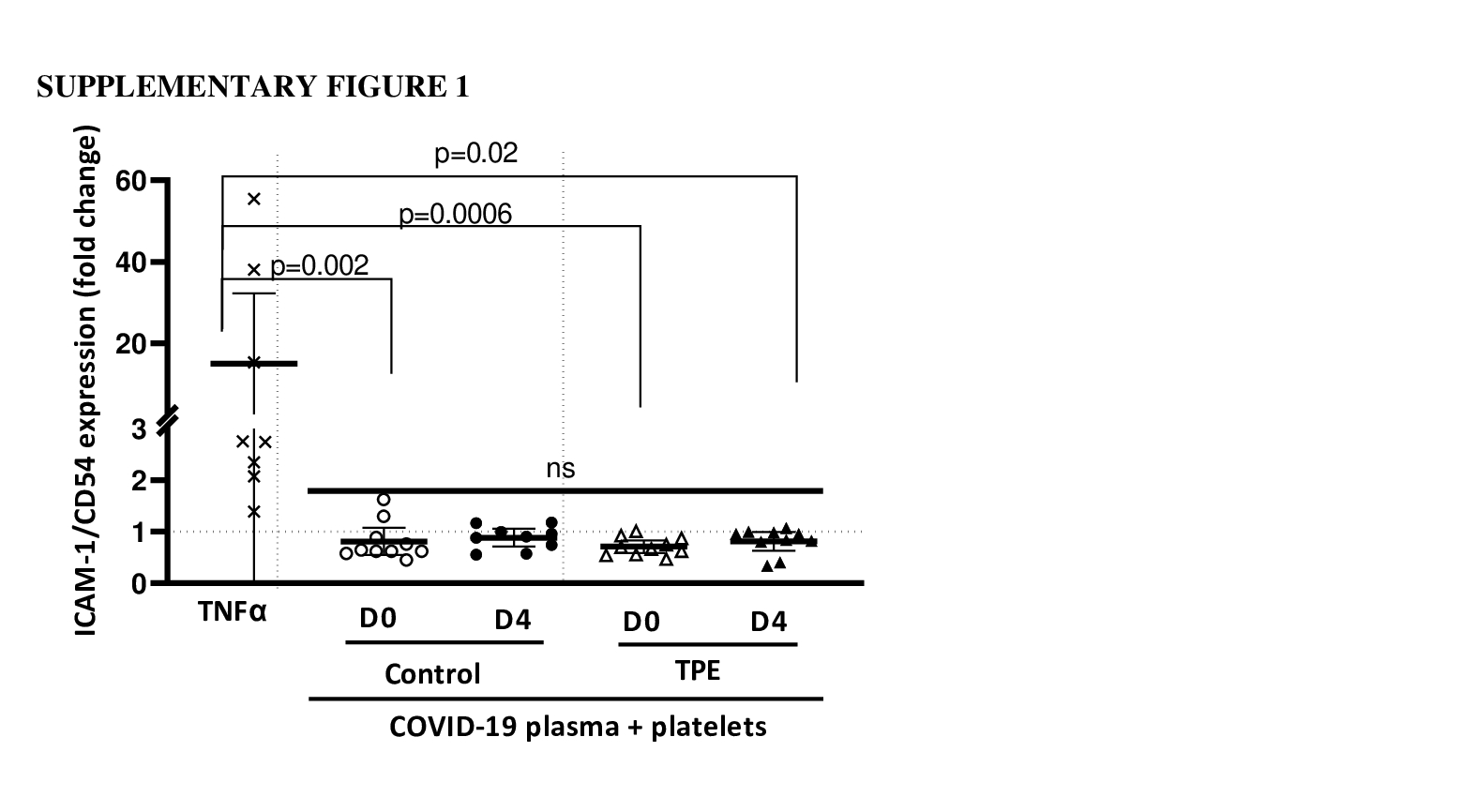

Supplement: Supplementary file 1 [file Image1.jpeg]

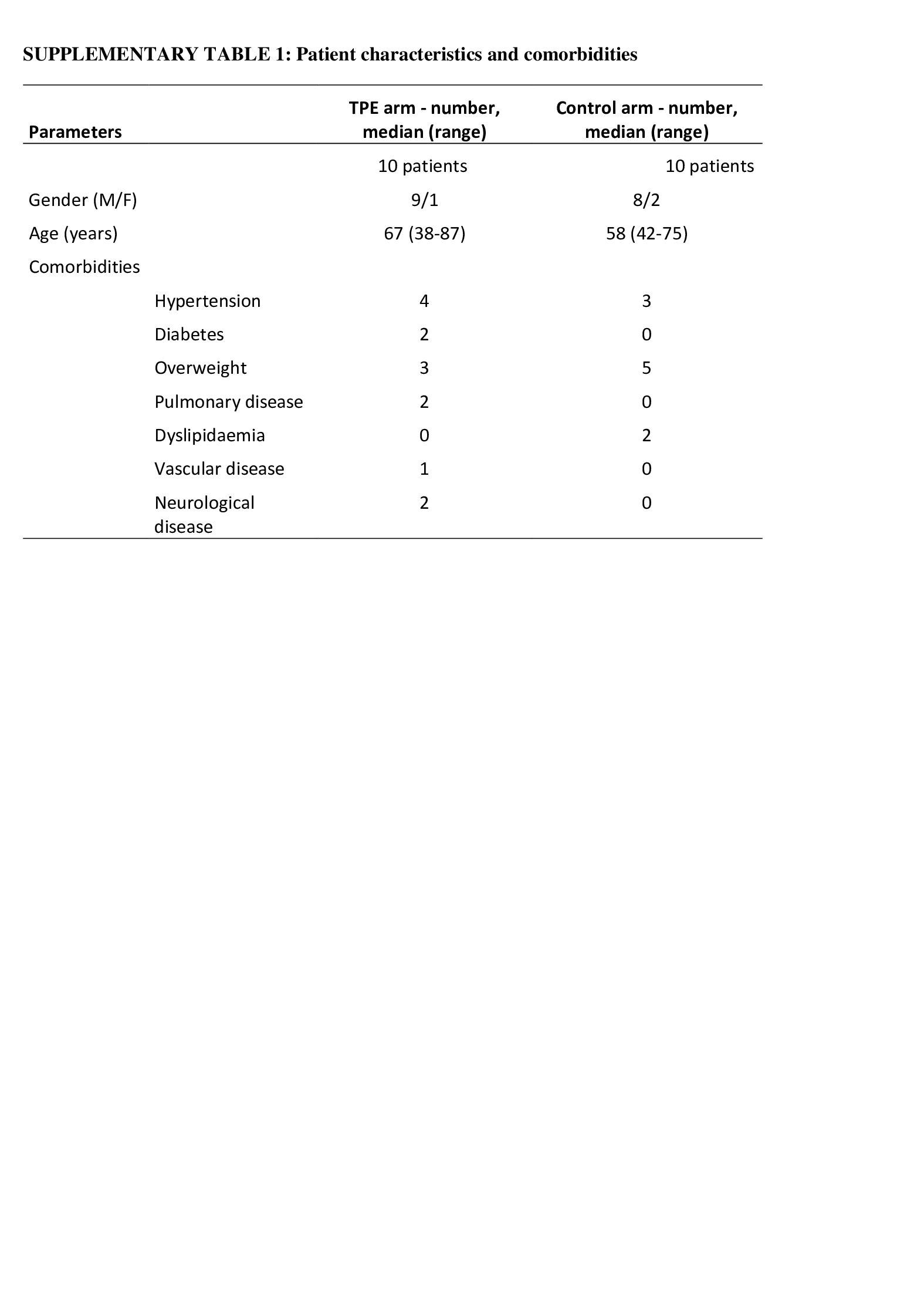

Supplement: Supplementary file 2 [file Image2.jpeg]
